# Heavy prenatal alcohol exposure and healthcare use during childhood and adolescence: a Danish nationwide cohort study 1997–2022

**DOI:** 10.1007/s10654-025-01280-3

**Published:** 2025-07-24

**Authors:** Julie Marie Winckler, Kathrine Kold Sørensen, Katrine Strandberg-Larsen, Christian Torp-Pedersen, Ulrik Schiøler Kesmodel, Mikkel Porsborg Andersen, Marcella Broccia

**Affiliations:** 1 Department of Cardiology, Nordsjaellands Hospital, 3400 Hillerød, Denmark; 2 Section of Epidemiology, Department of Public Health, University of Copenhagen, Copenhagen, Denmark; 3 Section of Biostatistics, Department of Public Health, University of Copenhagen, Copenhagen, Denmark; 4 Department of Obstetrics and Gynaecology, Aalborg University Hospital, Aalborg, Denmark; 5 Department of Clinical Medicine, Aalborg University, Aalborg, Denmark; 6 The Prehospital Center, Region Zealand, Denmark; 7 Department of Paediatrics and Adolescent Medicine, Zealand University Hospital, Roskilde, Denmark

**Keywords:** Alcohol use disorder, Fetal alcohol spectrum disorders, Epidemiology, Health care utilisation, Hospital contact, Preventive care

## Abstract

Children with heavy prenatal alcohol exposure may experience increased healthcare needs and access barriers. We aimed to quantify their healthcare utilisation patterns compared to all other Danish children in this nationwide cohort study of children born 1997–2020, followed until Dec 31, 2022. Data was derived from eight national registers. Heavy prenatal alcohol exposure was defined by hospital contacts with 100% alcohol-attributable diagnoses given to mother or child, redeemed prescriptions for drugs to treat alcohol dependence, or enrollment into treatment clinics, one year before or during pregnancy. We estimated age- and sex-specific rates and incidence rate ratios (IRRs) for use of somatic and psychiatric hospitals, and use of general practice (GP), and risk ratio (RR) for participation in the GP-based preventive child health programme. Of 1,457,962 children followed for 17,778,705 person-years, 5898 (0.4%) were heavily prenatal alcohol exposed. Exposed children had higher use of GP and hospitals compared to reference children, particularly notable for psychiatric hospital contacts with IRR for respectively ages 0–5, 6–11 and 12–18 years of 3.55 (2.98–4.24), 2.68 (2.41–2.98), and 2.19 (1.95–2.46); and for planned outpatient contacts 2.01 (1.92–2.10), 1.29 (1.21–1.37), and 1.20 (1.12–1.28). Despite higher healthcare use, participation in the preventive child health programme had a RR of 0.69 (95% CI 0.67–0.72) for exposed compared to reference children. The higher use of hospitals and GP, but lower participation in the child health programme underscores the long-term consequences and societal burden of heavy prenatal alcohol exposure, and leaves a need for awareness and adequate support to ensure health equity.

**Supplementary Information:**

The online version contains supplementary material available at 10.1007/s10654-025-01280-3.

## Introduction

Heavy prenatal alcohol exposure remains a key public health concern due to its potential adverse effects on fetal development leading to an increased risk of life-long physical, cognitive, and behavioural impairments [1]. Prenatal alcohol exposure can also lead to fetal alcohol spectrum disorders (FASD). The global prevalence of any alcohol use during pregnancy has been estimated at 9.8% [2], and the prevalence of FASD at 19.8 per 1000 children in the European Region [3]. Prevalences might be overestimates due to secular trends of lower levels of alcohol drinking in pregnancy [4], nonetheless they represent best available estimates and pregnant women often tend to conceal their alcohol use, and FASD is underdiagnosed. While heavy prenatal alcohol exposure is associated with increased child morbidity and mortality [5, 6], there are few studies on healthcare use among children with heavy prenatal alcohol exposure and existing studies primarily focus on selected populations. Some studies have found FASD associated with higher healthcare use [7, 8], while other studies have described barriers to health care for children with FASD, including lack of knowledge, complex needs, misinterpretation of the children’s behaviour, and caregiver incapacities [9, 10]. Children affected by prenatal alcohol exposure constitute a vulnerable group with both potential life-long impairments caused by alcohol and a high risk of co-occurring childhood adversities such as parental mental disorders, out of home care placement, neglect, and abuse [5, 11]. Patterns of healthcare use can highlight attention points for health care providers and inform policy efforts to promote health equity. This nationwide cohort study aimed to assess healthcare utilisation among children with heavy prenatal alcohol exposure compared to all other Danish children, using national registry data. To achieve a nuanced understanding of healthcare-seeking behaviour and system-level responsiveness, we aimed to: (1) quantify use across eight distinct healthcare services, including somatic and psychiatric hospitals, general practice (GP), and participation in the universal GP-based Danish child health programme; (2) describe age- and sex-specific rates in utilisation; (3) describe hospital admission characteristics, including length of stay, among alcohol-exposed children. These insights may help identify service gaps and guide targeted improvements in care for this vulnerable population.

## Methods

### Study design, participants, and setting

In this nationwide Danish register-based cohort study, we included all liveborn children from Jan 1, 1997 to Dec 31, 2020. Individuals were followed until age 18, emigration, death or Dec 31, 2021 for GP outcomes and Dec 31, 2022 for hospital outcomes. We excluded children of women migrating into Denmark one year before or during the pregnancy, children or mothers with unverified identities, and children registered as immigrants but born in Denmark. To study preventive child health programme participation, a subpopulation was formed, including children born 1997 to 2015 and residing in DK to 6 years of age.

The Danish healthcare system is free and publicly funded. Preventive Health Services for Children and Adolescents in Denmark include antenatal care, home visits by a community nurse in the first year of life, and seven scheduled exams in the nationwide preventive child health programme provided by a general practitioner within the first five years of life (at 5 weeks, 5 months, and 1–5 years of age), as well as a vaccination programme [12].

### Data sources

All Danish residents are assigned a personal identification number at birth or immigration for administrative purposes, enabling individual-level linkage of the national registries. We derived the study population and information on date of birth, gestational age, sex assigned at birth, parity, multiple births, and maternal age from the Danish Medical Birth Register [13]. Mother and child identities were verified by identification in and information on migrations, ethnicity and dates of death obtained from the Civil Registration System [14]. Data on maternal education was obtained from the Population Education Register [15].

Information on hospital contacts and diagnosis codes according to the International Classification of Disease revision 10 (ICD-10) was obtained from the Danish National Patient Register, which holds information on all contacts for non-psychiatric and psychiatric public hospitals since 1995 [16]. Information on medication was obtained from the Danish National Prescription Registry, which holds information on redeemed prescriptions at pharmacies since 1995 [17].

Data on enrollment into alcohol and substance use disorder treatment clinics was obtained from the Danish National Registry of Alcohol Treatment and the Danish Registry of Drug Abusers Undergoing Treatment, established in 2006 and 1996 respectively [18, 19].

Information on GP contacts was obtained from the Danish National Health Insurance Service Register, which records reimbursements to publicly funded non-hospital healthcare providers [20].

### Exposure

Heavy prenatal alcohol exposure was defined by presence of at least one of the following: (1) a hospital contact with a 100% alcohol-attributable diagnosis given to the mother during pregnancy or within one year prior to pregnancy; (2) a maternal redeemed prescription for drugs to treat alcohol dependence during pregnancy or within the year prior to pregnancy; (3) maternal enrollment during or within the year prior to pregnancy in alcohol treatment clinics or clinics for substance use disorder treatment if co-use of alcohol; (4) a hospital contact with a prenatal alcohol exposure attributable diagnosis given to the child at all times (Table S1). We used gestational age to define the period of pregnancy and missing information was replaced with 40 weeks of gestation. Gestational age was in Denmark determined by ultrasound biometry in pregnancy weeks 17–18 before 2005 and 11–14 after 2005 [21].

For accuracy, we included children born to mothers with alcohol-attributable conditions during or within the year prior to pregnancy, as many children with FASD remain undiagnosed [22]. We included the year prior to pregnancy since pre-pregnancy alcohol use is a strong risk factor for use during pregnancy [23, 24].

Of note, alcohol use is systematically assessed by the GP at the first antenatal visit, and individuals with self-reported problematic use are referred to extended hospital care where a 100% alcohol-attributable diagnosis is given.

Results are reported for exposed children compared to children not identified as exposed, hereafter termed reference children.

### Outcomes

Outcomes of interest were children´s utilisation of eight types of hospitals and GP contacts from age 0 to 18 years.

#### Hospital utilisation

Hospital use was divided into five types of services with psychical contacts: (1) Neonatal admissions, defined as contacts registered as admissions or as contacts with a duration longer than or equal to five hours, starting within the first 28 days of life and with the main medical speciality being paediatric and with a recorded diagnosis within the ICD–10 Chapter XVI “Certain conditions originating in the perinatal period” (P00–P96); (2) Acute contacts, defined as all acute and emergency hospital contacts independent of the duration of the contact; (3) Planned hospital admissions, defined as contacts registered as planned admissions or as planned contacts with a duration longer than or equal to five hours; (4) Planned outpatient hospital contacts, defined as contacts registered as planned outpatient contacts or as planned contacts with a duration shorter than five hours; and (5) Psychiatric hospital contacts, defined as all contacts to psychiatric hospitals or departments, irrespective of the contact type.

Contacts were, except for neonatal admissions, included from 29 days up to 18 years of age. All admissions were defined as inpatient stays not overlapping or adjacent to other inpatient stays in time; Transfers and readmissions within 24 h were merged. For contacts with missing end dates, the end date was set to the same day as the start date. There were modifications of the structure of the registers throughout the study period; our definitions attempt to account for this, see table S2 for details.

#### General practice (GP) utilisation

GP use was divided into three types of services: (1) GP consultations; (2) Participation in the seven preventive child health programme exams, and (3) GP additional services to standard consultations, such as clinical procedures, laboratory tests, telephone- and email-contacts. Vaccinations were not included. There were modifications of the structure of the register in 2005, for which year we removed obvious duplicate registrations. For the child health programme exams we allowed for only one registration per week and restricted to a maximum of seven exams (Table S2).

### Covariates

Covariates included were: Birth year; sex assigned at birth; maternal age at birth; maternal ethnicity categorised as Danish if one parent held citizenship and were born in Denmark or if no information was registered, as immigrant or as descendant of immigrants (table S3); highest achieved maternal educational level in the year prior to the birth categorised according to the International Standard Classification of Education, however with no information on education categorised as primary and lower secondary education (table S3); maternal parity categorised as status before birth of the index child as nulliparous or primi- and multiparous; multiple births; preterm birth defined as gestational age before 37 + 0 weeks; prenatal substance use exposure defined as a hospital contact with a substance-attributable diagnosis given to the newborn or mother or a maternal enrollment into substance use treatment one year before or during pregnancy (table S4); and maternal mental disorders defined as hospital contacts within two years of giving birth with a diagnosis in the ICD-10 chapter “Mental and behavioural disorders” (F00–F99), excluding diagnoses due to alcohol or substance use (table S1 and S4), or with the diagnosis “Mental disorders complicating pregnancy, childbirth and the puerperium” (O99.3B) (table S3). Covariates were selected based on prior literature and clinical relevance, reflecting factors associated with prenatal alcohol exposure and healthcare utilisation [5, 7–9, 11, 23].

### Statistical methods

For hospital and GP use, recurrent events were included to assess the burden of healthcare use. We report the total number of events, proportions of children with at least one contact, and unadjusted age- and sex-specific rates taking into account time at risk by censoring at death, emigration, age 18, or end of study period. Quartiles for length of stay are reported for planned hospital admissions and neonatal admissions. To estimate the incidence rate ratio (IRR) between groups, Generalised Estimating Equation (GEE) Poisson models with a logarithmic link were applied, accounting for non-independence of observations by incorporating an exchangeable within subject-observations correlation structure. Standard errors were calculated using robust sandwich estimates and p-values were obtained through the Wald test. IRRs were estimated for subgroups of early childhood (0–5 years), late childhood (6–11 years), and adolescence (12–18 years). Outcomes were adjusted for: (1) year, sex, and age of child (cubic spline due to non-linearity; age not included for neonatal admissions); (2) additionally maternal age, ethnicity, and education.

For the child health programme, we report quartiles for and proportions of children with participation in different numbers of exams. Risk ratio (RR) for participation in above five exams was estimated using log-binomial models, with above five chosen due to the reference group having a median of six exams. Outcomes were adjusted for (1) sex and birth year using a cubic spline due to non-linearity; (2) additionally maternal age, ethnicity, and education.

Supplementary analyses were conducted for all outcomes examining interactions between heavy prenatal alcohol exposure and respectively other prenatal substance use exposure and maternal mental disorders. Additionally, for hospital and GP outcomes, a supplementary analysis report on tests for sex-specific interactions and estimates for sex-specific subgroups.

Sensitivity analyses were conducted for all outcomes: (1) Restricting the study population to singleton first-born children to account for correlated observations; (2) restricting the study period to 2008 and onwards, corresponding to extended data availability from the National Register on Alcohol Treatment; (3) restricting the heavy alcohol exposure definition to solely include maternal diagnoses. Regarding the outcome of the child health programme, in the sensitivity analysis with a restricted study period, maternal age and birth year were categorised to avoid empty strata and ensure model fit. For the child health programme, an additional sensitivity analysis with restriction to children born at term, gestational age 39 + 0 to 40 + 6 weeks, were conducted. For hospital and GP outcomes, an additional sensitivity analysis applied an independent correlation structure in the GEE-models to check robustness of results.

R-software version 4.2.1 was used for data management and statistical analysis [25].

## Results

In total, 1,457,962 live-born children were followed from 1997 to 2022 with a mean total follow-up age of 12.3 years, after exclusion of 59,381 children (figure S1). Of these, 5898 children (0.4%) were identified as exposed. The subpopulation for the outcome of the preventive child health programme consisted of 1,124,326 (4496 [0.4%] exposed) children. Table S5 shows the total number of healthcare contacts and person-time for healthcare outcomes within sex and age groups.

For calculating start of pregnancy in regard to exposure time, 2.1% had missing information on gestational age and 0.25% of all hospital contacts had missing end-of-contacts. For the covariates of maternal education and ethnicity, no information was registered for 1.15% and 0.003% respectively.

Compared to reference children, children with heavy prenatal alcohol exposure had higher mortality (72 [1.2%] vs. 8175 [0.6%], *p* < 0.001) while fewer emigrated during the study period (142 [2.4%] vs. 68,802 [4.7%], *p* < 0.001).

Mothers of exposed children were more often adolescent mothers and had lower educational levels, fewer were immigrants or descendants of immigrants. More exposed children were preterm and first-borns compared to reference children, and fewer were multiples. Compared to reference children, more children were prenatally exposed to other substance use (27.1% vs. 0.4%) and had mothers with mental disorders (35.8% vs. 4.3%) (Table 1).

**Table 1:** Baseline characteristics of prenatally heavily alcohol exposed and reference children

		Reference children (*n* = 1,452,064)	Exposed children (*n* = 5898)
Sex	Girls	706,918 (48.7%)	2866 (48.6%)
	Boys	745,146 (51.3%)	3032 (51.4%)
Birth year	1997–2001	319,487 (22.0%)	1155 (19.6%)
	2002–2006	314,028 (21.6%)	1022 (17.3%)
	2007–2011	305,016 (21.0%)	1229 (20.8%)
	2012–2016	278,581 (19.2%)	1474 (25.0%)
	2017–2020	234,952 (16.2%)	1018 (17.3%)
Maternal ethnicity	Danish or no information registered	1,244,000 (85.7%)	5397 (91.5%)
	Immigrants	181,034 (12.5%)	425 (7.2%)
	Descendant of immigrants	27,030 (1.9%)	76 (1.3%)
Maternal age at birth, years	12–20	32,325 (2.2%)	956 (16.2%)
	21–30	745,772 (51.4%)	3007 (51.0%)
	31–40	648,292 (44.6%)	1795 (30.4%)
	41–61	25,675 (1.8%)	140 (2.4%)
Highest achieved maternal education prior to birth	Primary and lower secondary or no education registered	271,927 (18.7%)	3686 (62.5%)
	Upper secondary	560,741 (38.6%)	1574 (26.7%)
	Short cycle tertiary, bachelor or equivalent	432,939 (29.8%)	493 (8.4%)
	Master or equivalent, doctoral or equivalent	186,457 (12.8%)	145 (2.5%)
Multiple births		55,394 (3.8%)	157 (2.7%)
Parity	Nulliparous	630,682 (43.4%)	3540 (60.0%)
	Primi- and multiparous	786,895 (54.2%)	2226 (37.7%)
	Missing	34,487 (2.4%)	132 (2.2%)
Preterm birth (< GA 37 weeks)	Yes	94,449 (6.5%)	638 (10.8%)
	Missing	27,137 (1.9%)	124 (2.1%)
Maternal mental disorders		63,053 (4.3%)	2112 (35.8%)
Prenatal substance use exposure		5186 (0.4%)	1598 (27.1%)

**Fig. 1:**
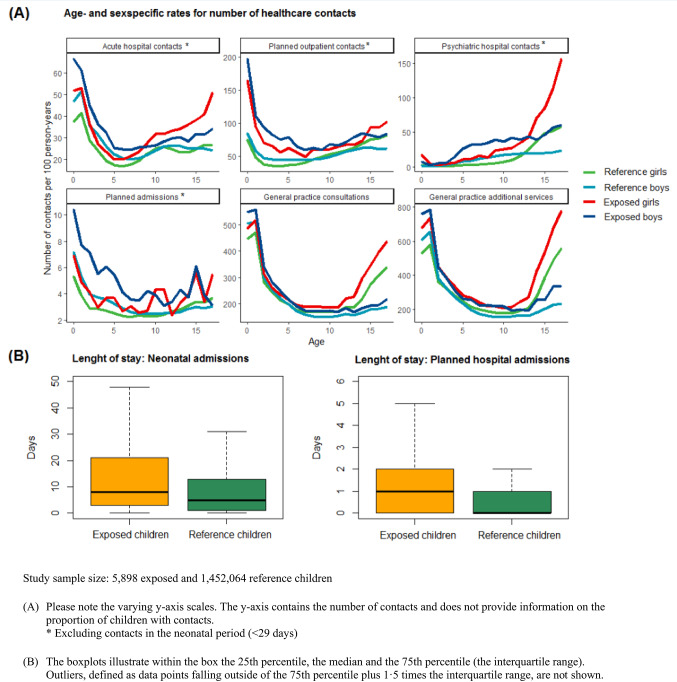
Patterns of healthcare use for children with heavy prenatal alcohol exposure and reference children: **A** Age- and sex-specific rates for number of healthcare contacts, **B** Distribution of hospital admissions lengths

Of the eight healthcare services assessed, Fig. 1A presents age- and sex-specific rates for six healthcare contacts. Rates were higher among children with heavy prenatal alcohol exposure compared to reference children for acute hospital contacts, planned outpatient hospital contacts, planned hospital admissions, psychiatric hospital contacts, GP consultations, and GP additional services. At different ages, boys and girls exhibited varying healthcare utilisation patterns. Sex-specific utilisation patterns among exposed children overall resembled that of reference children. Longer length of stay for planned hospital admissions and neonatal admissions were observed for exposed children compared to reference children (Fig. 1B).

For hospital outcomes, more exposed children than reference children had at least one contact, e.g. 23.2% vs. 12.5% had at least one neonatal admission (table S6). For GP outcomes, 99% of all children had at least one contact.

**Fig. 2:**
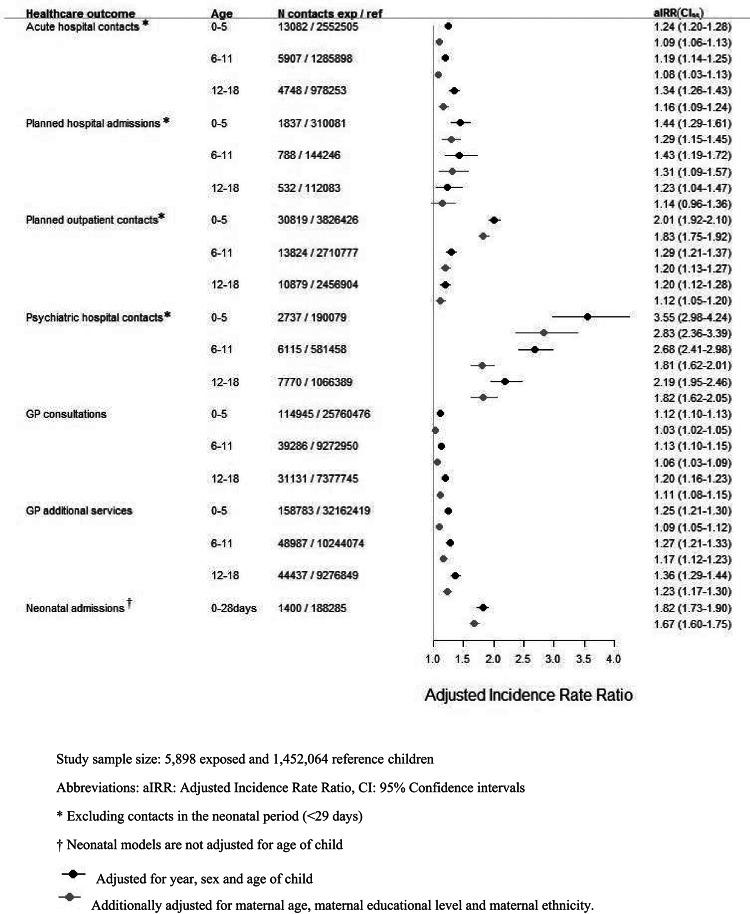
Incidence rate ratios for healthcare contacts among children with prenatal heavy alcohol exposure compared to reference children in age groups of 0–5, 6–11 and 12–18 years

For all hospital and GP outcomes and within all age groups, adjusted incidence rate ratio estimates suggested relative increases in the mean number of contacts for exposed children compared to reference children, with only one estimate for planned hospital admissions in ages 12–18 not being statistically significant. Estimates adjusted for year, sex, and age of child suggested relative increases between 19 and 101% in somatic hospital use, e.g. for ages 0–5 years, 6–11 years, and 12–18 years, respectively: IRR for exposed children compared to reference children for acute hospital contacts were 1.24 [95% CI 1.20–1.28], 1.19 [1.14–1.25], and 1.34 [1.26–1.43]; for planned outpatient contacts 2.01 [1.92–2.10], 1.29 [1.21–1.37], and 1.20 [1.12–1.28]; and for neonatal admissions 1.82 [1.73–1.90]). Likewise relative increases between 12 and 36% for GP use, and two to three times as many psychiatric hospital contacts for exposed children compared to reference children (aIRR 3.55 [2.98–4.24] for ages 0–5, 2.68 [2.41–2.98] for ages 6–11, and 2.19 [1.95–2.46] for ages 12–18 (Fig. 2). While estimates additionally adjusted for maternal age, ethnicity, and education were lower, patterns of higher healthcare use among exposed persisted.

**Fig. 3:**
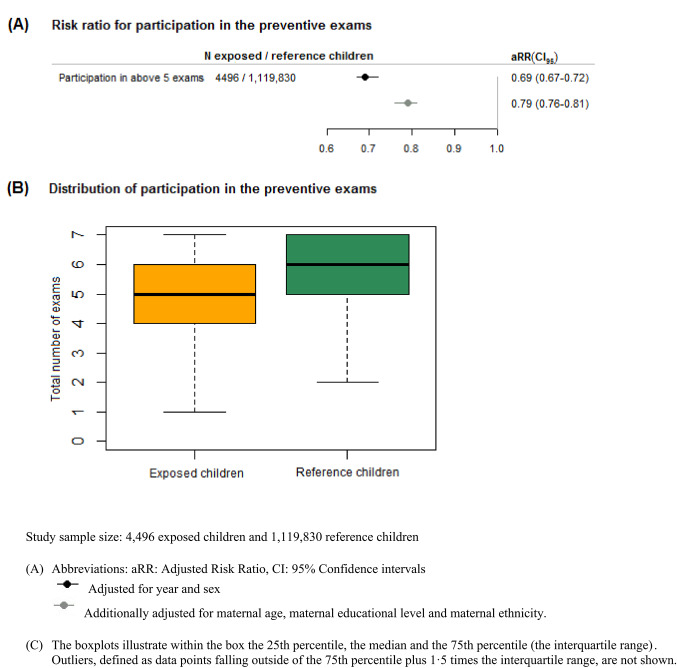
Association between heavy prenatal alcohol exposure and participation in the Danish preventive child health exams programme for children with full follow-up: **A** Risk ratios of participation in above five exams, **B** Distribution of participation

Exposed children had lower participation in the child health programme compared to reference children: 23.1% vs. 40.5% fully participated in the seven exams (table S7), and a median of five vs. six exams was observed (Fig. 3B). When adjusted for sex and birth year of child, exposed children were 31% less likely to have participated in above five out of seven exams (adjusted risk ratio 0.69, 95% CI 0.67–0.72) (Fig. 3A).

Supplementary analyses showed the higher use of health care to be evident for both children solely exposed to heavy prenatal alcohol exposure and for children with both heavy prenatal alcohol exposure and respectively other prenatal substance exposure or maternal mental disorders, when compared to children with no exposure of heavy prenatal alcohol exposure and respectively no other prenatal substance exposure or no maternal mental disorders (figure S2 and S3). Likewise, the lower participation in the child health programme was evident for both children with solely exposure and double exposures (figure S4). Higher healthcare use was found consistently for both boys and girls. Tests for interaction with sex were insignificant for all healthcare outcomes except neonatal admissions (figure S5).

Results were robust in regard to choice of correlation structure for the GEE-models: Sensitivity analysis with an independent correlation structure did not overall affect results, however results for planned hospital admissions age 12–18 became significant (figure S6). Main findings remained consistent in all sensitivity analyses with restriction to: singleton first-borns where estimates were slightly lower (figure S7, S10), extended data availability (figure S8, S10), and maternal exposure diagnoses (figure S9, S10).

Regarding extended data availability: Before incorporating a data source late in the study, the proportion of exposed children declined over time, but remained consistent afterward (Table 1).

## Discussion

Quantifying patterns of healthcare use among children with heavy prenatal alcohol exposure, we found higher use of hospitals and GP, and lower participation in the child health programme compared to reference children.

Findings of higher healthcare use are in line with previous studies on children with FASD [7, 8, 26]. Findings of lower participation in the child health programme remained consistent when restricting the analysis of participation to children born full-term, indicating that missed participation was not explained by the higher incidence of exposed children born preterm (figure S10).

Healthcare utilisation depends on both need and care-seeking behaviour. This study found that less than one in four exposed children (23.1%) attended all seven preventive child health programme exams, indicating that their higher use of hospital and GP services likely reflects greater health needs or delayed care seeking rather than a general healthcare-seeking prone behaviour. The lower uptake of the free-of-charge preventive child health programme suggests that, despite free access, non-financial barriers affect this vulnerable group. Therefore, higher healthcare use does not necessarily indicate optimal care. In settings with financial barriers, even greater disparities in preventive service use and health outcomes might be expected. With the cohort consisting of all live-born children in Denmark, this study also provides patterns of healthcare use for the general population of Danish children.

In accordance with literature describing accumulations of risk factors among children with prenatal alcohol exposure, we found exposed children more likely to have other prenatal substance use exposure (27.1% vs. 0.4%) and maternal mental disorders (35.8% vs. 4.3%). Supplementary analyses showed that both as single risk factors and as double exposures, heavy prenatal alcohol exposure, other prenatal substance use exposure, and maternal mental disorders were associated with patterns of higher healthcare use. This is in line with studies that have shown higher healthcare use among children with different risk profiles such as maternal mental- and alcohol use disorders [27], maternal alcohol and substance use disorders [28, 29], and among children with a high level of childhood adversities [30]. We also found that 16.4% of exposed children had mothers younger than 21 years of age at time of birth compared to 2.4% of reference children, which underlines the importance of preconception care.

Nationwide registry data enabled this large-scale study, supplementing existing studies by including different healthcare services and ensured no susceptibility to selective inclusion or recall bias. Danish registers are generally believed to be of high validity and completeness. FASD is overlooked, and since assessment of prenatal alcohol exposure relies on self-reports prone to social desirability bias, underreporting is likely. To improve identification and reduce misclassification, we assessed heavy prenatal alcohol exposure based on both maternal and child diagnoses, an approach previously used in registry-based studies [5] and inspired by work of O’Leary and colleagues [24]. While misclassification remains possible, it would likely bias results towards the null and thus reflects a conservative, best-informed estimate in a Danish context.

The exposure definition holds no information on timing, frequency, or quantity of alcohol intake, and represents only the most heavy use which is a small fraction of any alcohol use during pregnancy. Data on privately funded or anonymous enrollments in alcohol or substance use treatment clinics are incomplete. Data on alcohol clinic enrollments covers the years from 2006 and forwards, however sensitivity analysis estimates aligned with main estimates. We did not include information on maternal smoking in this study, which is however expected to co-occur with prenatal alcohol exposure. We chose to estimate results with adjustment for two sets of covariates: one adjusted for age, sex and year, and one additionally adjusted for sociodemographic factors. Several additional covariates, including sociodemographic characteristics, are likely part of a broader risk context associated with prenatal alcohol exposure. To reflect differences in healthcare use associated with heavy prenatal alcohol exposure, careful consideration of model adjustments is essential to avoid bias from overadjustment. Therefore, we consider the lightly adjusted models more appropriate for capturing overall patterns and also believe these most relevant from a clinical and public health perspective. Independently of the chosen adjustment, patterns of higher healthcare use among exposed children persisted.

This study shows that despite a comprehensive nationwide free preventive child health programme, the programme which requires self-referral might not be well-suited for caregivers in vulnerable families and therefore fail to capture the possibly most vulnerable children and families. Overall, these findings highlight the long-term individual consequences and societal burden of heavy prenatal alcohol exposure and indicate a need for adequate support to ensure health equity and child well-being for a vulnerable group of children.

## Electronic supplementary material

Below is the link to the electronic supplementary material.

Supplementary Material 1
