# Supplementary material for: Heavy prenatal alcohol exposure and healthcare use during childhood and adolescence: a Danish nationwide cohort study 1997–2022
Source: Eur J Epidemiol. 2025 Jul 24;40(9):1095–104. doi: 10.1007/s10654-025-01280-3 (PMC12537608; doi:10.1007/s10654-025-01280-3)
Supplement: Supplementary file 1 — Supplementary Material 1 [file 10654_2025_1280_MOESM1_ESM.docx]

**Supplementary material:** Heavy prenatal alcohol exposure and healthcare use during childhood and adolescence: a Danish nationwide cohort study 1997-2022

**Authors**: Julie Marie Winckler, MscPH (1), Kathrine Kold Sørensen, PhD (1), Katrine Strandberg-Larsen, PhD (2), Prof Christian Torp-Pedersen, DrMedSci (1,3), Prof Ulrik Schiøler Kesmodel, PhD (4,5), Mikkel Porsborg Andersen, PhD (1,6), Marcella Broccia, MD (1,4,7)

1: Department of Cardiology, Nordsjaellands Hospital, Hillerød, Denmark

2: Section of Epidemiology, Department of Public Health, University of Copenhagen, Copenhagen, Denmark

3: Section of Biostatistics, Department of Public Health, University of Copenhagen, Copenhagen, Denmark

4: Department of Obstetrics and Gynaecology, Aalborg University Hospital, Aalborg, Denmark

5: Department of Clinical Medicine, Aalborg University, Aalborg, Denmark

6: The Prehospital Center, Region Zealand, Denmark

7: Department of Paediatrics and Adolescent Medicine, Zealand University Hospital, Roskilde, Denmark

**Correspondence to**: Julie Marie Winckler, Department of Cardiology, Nordsjaellands Hospital, Hillerød, 3400, Denmark. Mail: [julie_marie@live.dk](mailto:julie_marie@live.dk).

**Table S1: Specification of the defining criteria for heavy prenatal alcohol exposure**

|  | **Conditions and drugs** | **ICD-10 and ATC codes** [References] |
| --- | --- | --- |
| **Maternal 100% alcohol-attributable diagnoses** | Alcohol-induced pseudo-Cushing's syndrome | E244 [2] |
|  | Alcoholic niacin deficiency [pellagra] | E529A ^* [2]^ |
|  | Mental and behavioural disorders due to use of alcohol | F10, F10.1–F10.9 [1]  *(excl. F100, F1000-1007)* |
|  | Degeneration of nervous system due to alcohol | G31.2 [1] |
|  | Alcoholic polyneuropathy | G62.1 [1] |
|  | Alcoholic myopathy | G72.1 [1] |
|  | Alcoholic cardiomyopathy | I42.6 [1] |
|  | Alcoholic gastritis | K29.2 [1] |
|  | Alcohol liver disease | K70 [1] |
|  | Alcohol-induced acute pancreatitis | K85.2 [1] |
|  | Alcohol-induced chronic pancreatitis | K86.0 [1] |
|  | Alcohol abuse counselling and surveillance | Z71.4 [2] |
|  | Alcohol use | Z72.1 [2] |
|  | Mental and behavioural disorders due to use of alcohol: acute intoxication | F10.0 [1] *(incl. F100, F1000-1007 but excl. F10, F101-109)* |
|  | Finding of alcohol in blood | R78.0 [2] |
|  | Disulfiram-alcohol reaction | T50.0A ^*^ [2] |
|  | Toxic effects of alcohol | T51.0-T51.9 [2] |
|  | Accidental poisoning by and exposure to alcohol | X45 [1] |
|  | Intentional self-poisoning by and exposure to alcohol | X65 [1] |
|  | Maternal care for (suspected) damage to fetus from alcohol | O354 [1] |
|  | Alcohol use during pregnancy | Z35.8M10 ^*^ [2] |
|  | Prenatal alcohol exposure | Z07.1A ^†* [2]^ |
| **Maternal alcohol-treatment by redeemed prescriptions** | Disulfiram | N07BB01 [2] |
|  | Acamprosat | N07BB03 [2] |
|  | Naltrexon | N07BB04 [2] |
|  | Nalmefen | N07BB05 [2] |
| **Maternal alcohol-treatment by enrollment into clinics** | Enrolled in alcohol treatment registered in the NRAT | |
|  | Enrolled in substance use treatment with co-use of alcohol registered in the RDAT | |
| **100% alcohol-attributable diagnoses given to child** | Fetus and newborn affected by maternal use of alcohol | P04.3 [1] |
|  | Prenatal alcohol exposure | Z07.1A ^†* [2]^ |
|  | Fetal alcohol Syndrome (FAS) | Q86.0 [1] |
|  | Fetal alcohol spectrum disorder (FASD) ^§^ | Q86.3 ^* [3]^ |
| *Abbreviations*: *ICD-10 codes*, International Classification of Diseases, tenth Edition; *ATC codes*, Anatomic Therapeutic Chemical Codes, *NRAT*: The Danish National Registry of Alcohol Treatment, *RDAT*: The Danish Registry of Drug Abusers Undergoing Treatment.  References: See list in the end of this document. Diagnoses are defined by pre-existing lists last checked for updates 17.01.2023.  * National modified ICD-10 codes  † Diagnosis is primarily given to child, secondarily to the mother and therefore included twice  § The FASD-diagnosis was first introduced in Denmark from 2021 and forwards, and very few children in this study are diagnosed with FASD in 2021-2022. | | |

**Table S2: Outcome definitions based on data in the registers**

| **Outcomes** | | **Definition based on *variables*** |
| --- | --- | --- |
| **Hospital contacts *** | Emergency contacts (somatic) | DNPRv.2: Defined by (*pattype*=3) or (*pattype*=0,1,2 and *indm*=1). ^†^  DNPRv.3: Defined by (*prioritet*=ATA1 and *kontakttype*=ALCA00). |
|  | Planned admissions (somatic) | DNPRv.2: Defined by (*pattype*=0,1 and *indm*=2,NA,9).  DNPRv.3: Defined by (*prioritet*=ATA2,ATA3 and *kontakttype*=ALCA00 and a duration longer than or equal to 5 hours).  All admissions defined as non-overlapping and non-adjacent. ^‡^ |
|  | Planned outpatient contacts (somatic) | DNPRv.2: Defined by (*pattype*=2 and *indm*=2,NA,9) and merged with outpatient visits registered in the table *t_bes.*  DNPRv.3: Defined by (*prioritet*=ATA2,ATA3 and *kontakttype=*ALCA00 and duration shorter than 5 hours). |
|  | Psychiatric contacts | Defined by the department’s medical speciality being ‘psychiatry’ or ‘child- and youth-psychiatry’. A psychiatric contact is either a psychiatric admission (planned/emergency), or a shorter outpatient contact (planned/emergency), following the same definitions and data-management as applied to the somatic hospital contacts. ^‡^  For DNPRv.2: Merged with outpatient visits registered in the table *t_pers.* |
|  | Neonatal admissions | DNPRv.2: Defined by (*pattype*=0,1 and *spec*=80 and an diagnosis within the ICD-10 chapter P and the contact starting within day 0-28 of life).  DNPRv.3: Defined by (*kontakttype*=ALCA00 and *hovedspeciale_ans*=pædiatri and a duration longer than or equal to 5 hours and an diagnosis within the ICD-10 chapter P and the contact starting within day 0-28 of life). |
| **General practice contacts**^§^ | Standard consultations | Standard physical consultations, value *speciale* 800101  Services are registered weekly: Exact dates were set as Friday in the corresponding week. |
|  | Preventive child health exams | Values *speciale* 808200-808217. |
|  | Other services | All other services than the above within s*peciale* with first two digits 80, excluding all services covering vaccinations or additional services relating to vaccinations (8083XX-8089XX).  Services are registered weekly: Exact dates were set as Friday in the corresponding week. |
| * Data source: The Danish National Patient Register (DNPR), version 2 until 2019 (DNPRv.2) and version 3 from 2019 and onwards (DNPRv.3)  † Emergency department contacts were in DNPRv.2 defined by (*pattype*=3) before 2014; and (*pattype*=2 & *indm*=1) after 2014.  ‡ Transfers between departments or hospitals and readmissions within 24 hours were merged to one hospitalisation except for transfers between psychiatric and non-psychiatric departments or hospitals.  § Data source: The Danish National Health Insurance Service Register. | | |

**Table S3: Definition of covariates derived from the different registers**

| **Covariate** | **Registers** | **Definition** |
| --- | --- | --- |
| **Maternal mental disorders** | The Danish National Patient Register | Diagnoses in the ICD-10 *Chapter V: Mental and behavioural disorders*:  F00-F09, F20-F99 (excluding *disorders due to psychoactive substance use:* F10-F19) and the Danish national modified ICD-10 diagnosis *Mental disorders complicating pregnancy, childbirth, and the puerperium*: 099.3B |
| **Immigration** | The Danish Civil Registration System | Based on the variable *IE_type* (1=Danish, 2=immigrants, 3=descendants of immigrants) (Please note: Children in this study are born in Denmark and can therefore not be immigrants) |
| **Maternal education** | The Danish Population Education Register | Based on the variable *udd_niveau_k* and categorised according to the International Standard Classification of Education (ISCED) [7]: Primary and lower secondary (ISCED 0=Early childhood education,  ISCED 1=Primary education, ISCED 2=Lower secondary education or second stage of basic education); Upper secondary **(**ISCED 3=Upper secondary education); Short cycle tertiary, bachelor or equivalent (ISCED 5=Short-cycle tertiary education, ISCED 6=Bachelor’s or equivalent level); Master or equivalent and Doctoral or equivalent (ISCED 7=Master's or equivalent level, ISCED 8=Doctoral or equivalent level). |
| Abbreviations: *ICD-10*: International Classification of Diseases, tenth Edition. *ICD-8*: International Classification of Diseases, eight Edition. *ISCED*: International Standard Classification of Education [7]  References: See list in the end of this document. | | |

**Table S4: Summary of international classification of disease, tenth edition used to define the covariate ‘Prenatal exposure to substance use’**

| **Conditions attributed to substance use** | **ICD-10 codes [References]** |
| --- | --- |
| Mental and behavioural disorders because of misuse of drugs | F11–F16, F18, F19 [4, 6] |
| Maternal care for damage to the fetus by substance use | O35.5A ^*^ |
| Findings of drugs and other substances, not normally found in blood | R78.1-R78.4 [4] |
| Fetus and newborn affected by maternal use of drugs of addiction | P04.4 ^† [4]^ |
| Neonatal withdrawal symptoms from maternal use of drugs of addiction | P96.1 ^†^ [4] |
| Poisoning by narcotics and psychodysleptics | T40 [4-5] |
| Poisoning by psychostimulants with potential for use disorder | T43.6A-C ^*^[4-5] |
| Intentional self-poisoning by and exposure to narcotics and psychodysleptics [hallucinogens], not elsewhere classified | X62 |
| Prenatal exposure to substance use | Z071B1- Z071B7 ^*†^ |
| Substance use during pregnancy | Z35.8M11 - Z35.8M17^*^ |
| Substance use | Z72.2 |
| Abbreviations: *ICD-10 codes*, International Classification of Diseases, tenth Edition.  References: See list in the end of this document.  * National modified ICD-10 codes  † Diagnoses given to the newborn | |

**
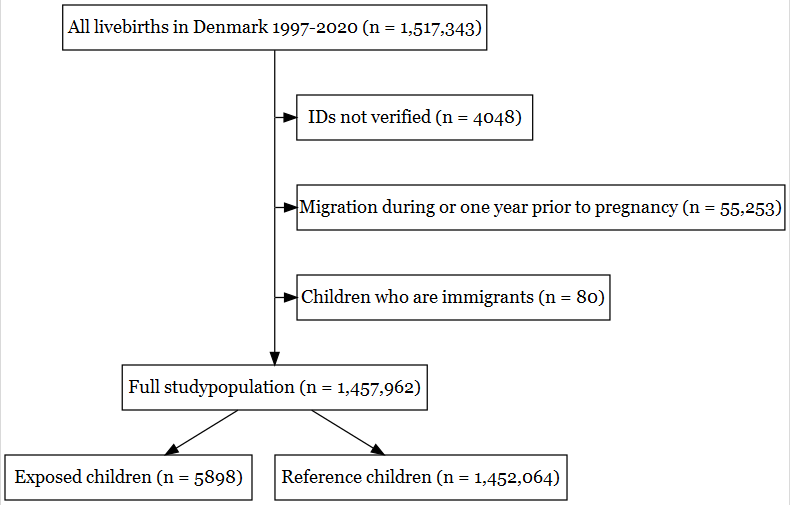
**

**Figure S1: Flowchart of the study population**

**Table S5: Number of healthcare contacts and person-time for healthcare outcomes for children with heavy prenatal alcohol exposure and reference children, within sex and age**

| **Healthcare outcome** | **Sex** | **Age** | **Exposure** | **Number of contacts** | **Person-time (years)** |
| --- | --- | --- | --- | --- | --- |
| **Acute hospital contacts** | Boys | 0-5 | Reference | 1,461,929 | 4,041,701.5 |
|  |  |  | Exposed | 7496 | 16,646.8 |
|  |  | 6-11 | Reference | 676,858 | 3,061,205.5 |
|  |  |  | Exposed | 3064 | 11,895.5 |
|  |  | 12-18 | Reference | 510,091 | 2,000,481.0 |
|  |  |  | Exposed | 2232 | 7251.9 |
|  | Girls | 0-5 | Reference | 1,090,576 | 3,822,883.2 |
|  |  |  | Exposed | 5586 | 15,535.8 |
|  |  | 6-11 | Reference | 609,040 | 2,895,778.7 |
|  |  |  | Exposed | 2843 | 11,165.0 |
|  |  | 12-18 | Reference | 468,162 | 1,887,553.7 |
|  |  |  | Exposed | 2516 | 6606.2 |

| **Planned hospital admissions** | Boys | 0-5 | Reference | 182,124 | 4,016,337.0 |
| --- | --- | --- | --- | --- | --- |
|  |  |  | Exposed | 1167 | 16,476.4 |
|  |  | 6-11 | Reference | 78,453 | 3,030,042.1 |
|  |  |  | Exposed | 440 | 11,715.5 |
|  |  | 12-18 | Reference | 55,205 | 1,969,297.1 |
|  |  |  | Exposed | 289 | 7071.6 |
|  | Girls | 0-5 | Reference | 127,957 | 3,776,971.6 |
|  |  |  | Exposed | 670 | 15,250.7 |
|  |  | 6-11 | Reference | 65,793 | 2,843,351.7 |
|  |  |  | Exposed | 348 | 10,875.0 |
|  |  | 12-18 | Reference | 56,878 | 1,834,972.7 |
|  |  |  | Exposed | 243 | 6317.7 |

| **Planned outpatient contacts** | Boys | 0-5 | Reference | 2,152,602 | 3,999,038.0 |
| --- | --- | --- | --- | --- | --- |
|  |  |  | Exposed | 17,626 | 16,552.2 |
|  |  | 6-11 | Reference | 1,405,364 | 3,021,866.3 |
|  |  |  | Exposed | 7446 | 11,799.5 |
|  |  | 12-18 | Reference | 1,181,745 | 1,962,528.1 |
|  |  |  | Exposed | 5641 | 7155.8 |
|  | Girls | 0-5 | Reference | 1,673,824 | 3,771,348.3 |
|  |  |  | Exposed | 13,193 | 15,451.5 |
|  |  | 6-11 | Reference | 1,305,413 | 2,846,941.2 |
|  |  |  | Exposed | 6378 | 11,104.7 |
|  |  | 12-18 | Reference | 1,275,159 | 1,840,085.7 |
|  |  |  | Exposed | 5238 | 6546.1 |

| **Psychiatric hospital contacts** | Boys | 0-5 | Reference | 130,221 | 4,018,305.1 |
| --- | --- | --- | --- | --- | --- |
|  |  |  | Exposed | 1610 | 16,231.6 |
|  |  | 6-11 | Reference | 423,242 | 3,031,580.7 |
|  |  |  | Exposed | 4107 | 11,479.4 |
|  |  | 12-18 | Reference | 392,946 | 1,970,991.1 |
|  |  |  | Exposed | 3181 | 6840.2 |
|  | Girls | 0-5 | Reference | 59,858 | 3,775,251.8 |
|  |  |  | Exposed | 1127 | 14,930.8 |
|  |  | 6-11 | Reference | 158,216 | 2,841,666.8 |
|  |  |  | Exposed | 2008 | 10,561.9 |
|  |  | 12-18 | Reference | 673,443 | 1,833,797.3 |
|  |  |  | Exposed | 4589 | 6011.5 |

| **GP consultations** | Boys | 0-5 | Reference | 1,3682,760 | 4,012,650.6 |
| --- | --- | --- | --- | --- | --- |
|  |  |  | Exposed | 61,578 | 16,400.2 |
|  |  | 6-11 | Reference | 4,557,446 | 2,928,832.0 |
|  |  |  | Exposed | 19,395 | 11,096.6 |
|  |  | 12-18 | Reference | 3,128,670 | 1,849,151.1 |
|  |  |  | Exposed | 12,441 | 6639.4 |
|  | Girls | 0-5 | Reference | 12,077,716 | 3,809,484.4 |
|  |  |  | Exposed | 53,367 | 15,379.3 |
|  |  | 6-11 | Reference | 4,715,504 | 2,783,020.7 |
|  |  |  | Exposed | 19,891 | 10,511.8 |
|  |  | 12-18 | Reference | 4,249,075 | 1,756,582.5 |
|  |  |  | Exposed | 18,690 | 6099.1 |

| **GP additional services** | Boys | 0-5 | Reference | 17,046,617 | 4,011,675.4 |
| --- | --- | --- | --- | --- | --- |
|  |  |  | Exposed | 83,327 | 16,400.0 |
|  |  | 6-11 | Reference | 4,946,465 | 2,928,513.7 |
|  |  |  | Exposed | 24,891 | 11,096.5 |
|  |  | 12-18 | Reference | 3,521,187 | 1,848,860.0 |
|  |  |  | Exposed | 16,778 | 6639.4 |
|  | Girls | 0-5 | Reference | 15,115,802 | 3,808,340.3 |
|  |  |  | Exposed | 75,456 | 15,379.3 |
|  |  | 6-11 | Reference | 5,297,609 | 2,782,789.3 |
|  |  |  | Exposed | 24,096 | 10,511.8 |
|  |  | 12-18 | Reference | 5,755,662 | 1,756,399.9 |
|  |  |  | Exposed | 27,659 | 6099.1 |

| **Neonatal admissions** | Boys | 0-28days | Reference | 107,719 | 57,157.6 |
| --- | --- | --- | --- | --- | --- |
|  |  |  | Exposed | 760 | 233.3 |
|  | Girls | 0-28days | Reference | 80,566 | 54,214.3 |
|  |  |  | Exposed | 640 | 220.1 |

**Table S6: The proportion of children with at least one healthcare contact among children with heavy prenatal alcohol exposure and reference children**

| **Healthcare outcome** | **Percentage with at least one contact** | |  |
| --- | --- | --- | --- |
|  | **Reference children** | **Exposed children** | |
| Acute hospital contacts | 81.5% | 84.7% |  |
| Planned hospital admissions | 18.3% | 24.2% |  |
| Planned outpatient contacts | 74.0% | 85.1% |  |
| Psychiatric hospital contacts | 9.4% | 22.5% |  |
| Neonatal admissions | 12.5% | 23.2% |  |
| General practice consultations | 99.2% | 99.2% |  |
| General practice additional services | 99.0% | 99.1% |  |

**Table S7: Participation in the seven preventive child health exams for children with heavy prenatal alcohol exposure compared to reference children, among the subpopulation of children with full follow-up**

|  | **Reference children**  n=1,124,326^*^ | **Exposed children**  n=4496* |
| --- | --- | --- |
| Full participation (7 or above exams) | 40.5 % | 23.1 % |
| Participation in 6 or above exams | 67.8 % | 47.0 % |
| Participation in 5 or above exams | 84.0 % | 70.0 % |
| Participation in 4 or above exams | 93.0 % | 85.5 % |
| Participation in 3 or above exams | 97.2 % | 94.3 % |
| Participation in 2 or above exams | 98.9 % | 98.2 % |
| Participation in 1 or above exams | 99.7 % | 99.6 % |
| No participation in the preventive child health exams | 0.3 % | - 1. % |
| ** Study sample size: 1,124,326* | | |


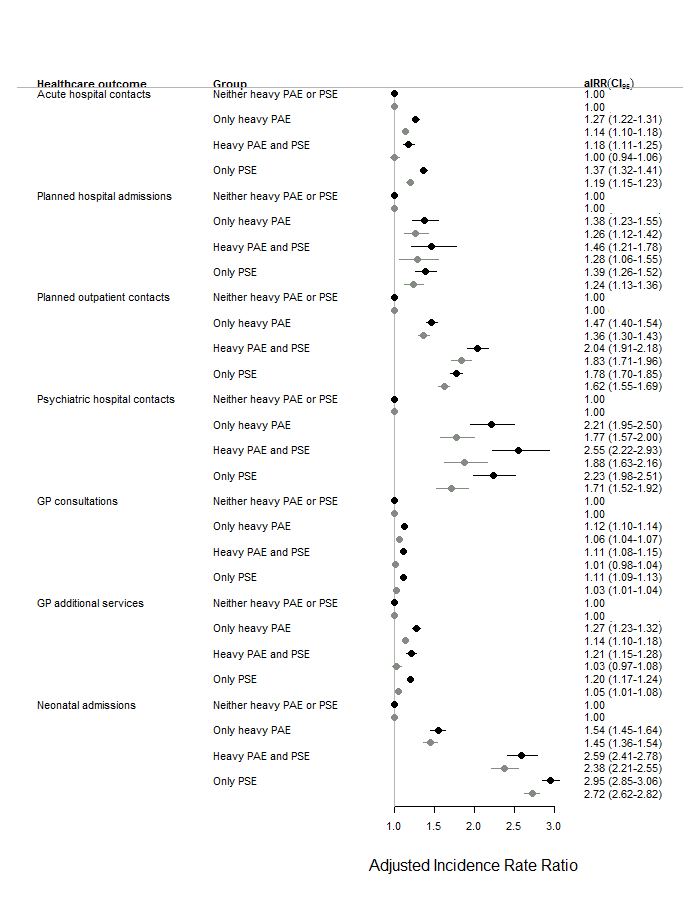
**Figure S2: Incidence rate ratios for number of healthcare contacts in groups of children with no heavy prenatal alcohol exposure (PAE) and no other prenatal substance use exposure (PSE) as the reference, compared to children with solely heavy prenatal alcohol exposure, to children with both heavy prenatal alcohol exposure and other prenatal substance use exposure, and to children with solely other prenatal substance use exposure**

Abbreviations: aIRR: Adjusted Incidence Rate Ratio, CI: 95% Confidence intervals

* Excluding contacts in the neonatal period (<29 days)

† Neonatal models are not adjusted for age of child


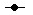
Black estimate is adjusted for year, sex and age of child


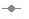
Grey estimate is additionally adjusted for maternal age, maternal educational level and maternal ethnicity.


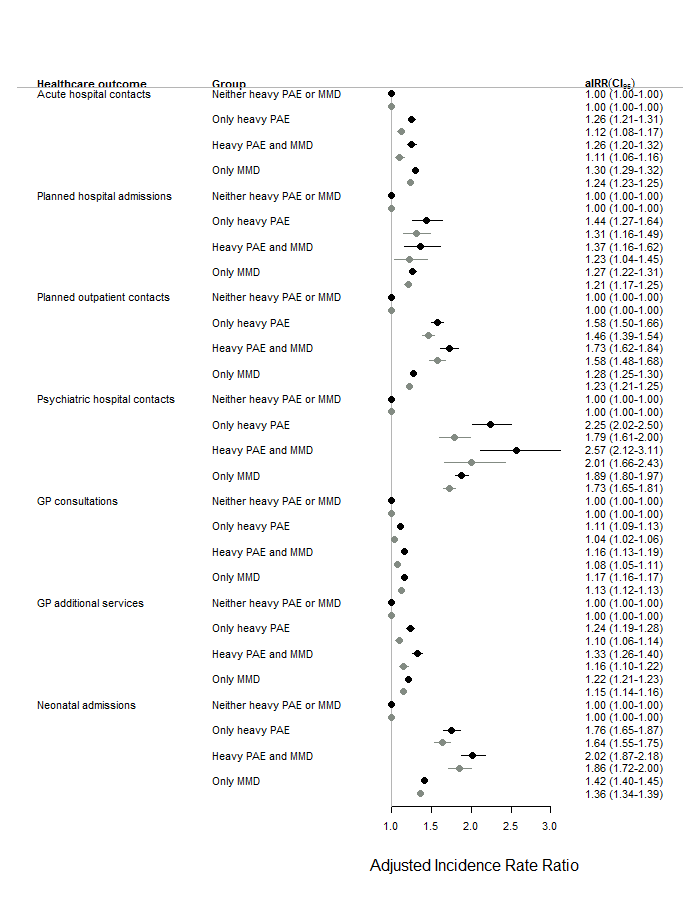


**Figure S3: Incidence rate ratios for number of healthcare contacts in groups of children with no heavy prenatal alcohol exposure (PAE) and no maternal mental disorders (MMD) as the reference, compared to children with solely heavy prenatal alcohol exposure, to children with both heavy prenatal alcohol exposure and maternal mental disorders, and to children with solely maternal mental disorders**

Abbreviations: aIRR: Adjusted Incidence Rate Ratio, CI: 95% Confidence intervals

* Excluding contacts in the neonatal period (<29 days)

† Neonatal models are not adjusted for age of child


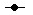
Black estimate is adjusted for year, sex and age of child


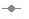
Grey estimate is additionally adjusted for maternal age, maternal educational level and maternal ethnicity.


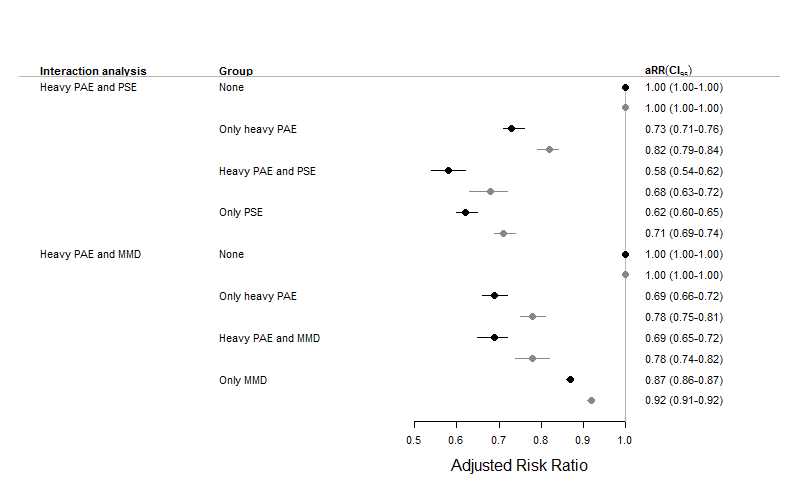


**Figure S4: Risk ratios for participation in the Danish preventive child health examination programme in groups of children with no heavy prenatal alcohol exposure (PAE) and respectively no other prenatal substance use exposure (PSE) or no maternal mental disorders (MMD) as the reference, compared to children with solely heavy prenatal alcohol exposure, to children with both heavy prenatal alcohol exposure and respectively other prenatal substance use exposure or maternal mental disorders, and to children with solely other prenatal substance use exposure or maternal mental disorders**

Study sample size: 4496 exposed children and 1,119,830 reference children

Abbreviations: aRR: Adjusted Risk Ratio, CI: 95% Confidence intervals


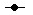
Black estimate is adjusted for year and sex


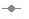
Grey estimate is additionally adjusted for maternal age, maternal educational level and maternal ethnicity.

**
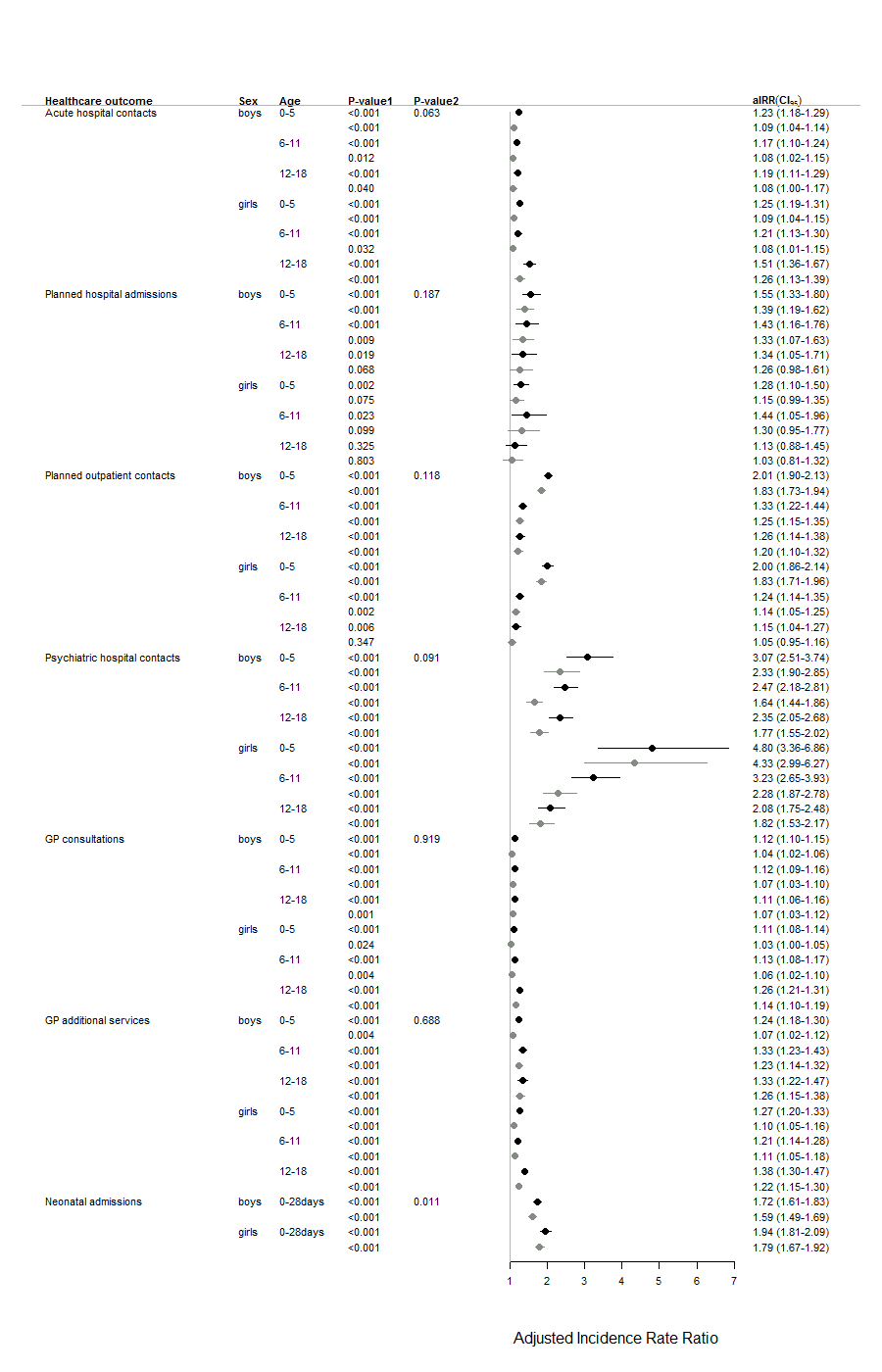
**
**Figure S5: Incidence rate ratios for healthcare contacts among children with heavy prenatal alcohol exposure compared to reference children within subgroups of sex and age, and with additional p-values from test of interaction between sex and heavy prenatal alcohol exposure**

**P-value1:** Test of significance in models within subgroups of sex and age

**P-value2:** Test for interaction between exposure and sex in models not stratified on age. P-values from models adjusted for year, sex and age are shown. P-values from models additionally adjusted for maternal age, education and ethnicity are not shown here but were for all important decimals identical.


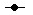
Black estimate is adjusted for year, sex and age of child


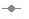
Grey estimate is additionally adjusted for maternal age, maternal educational level and maternal ethnicity.

**
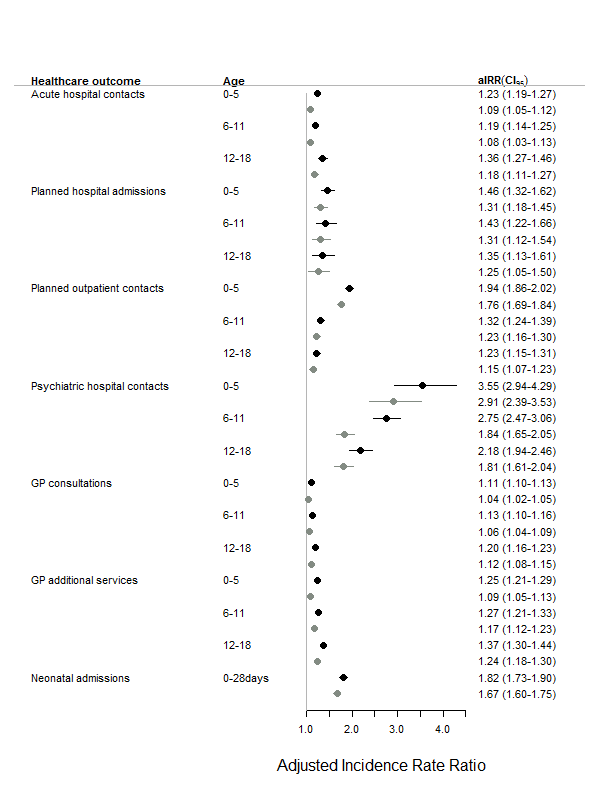
**

**Figure S6: Sensitivity analysis changing choice of correlation structure from exchangeable to independent in the GEE-models [8-9]: Incidence rate ratios for healthcare contacts among children with prenatal heavy alcohol exposure compared to reference children**

Study sample size: 5898 exposed, 1,457,962 reference children

References: See list in the end of this document.


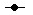
Black estimate is adjusted for year, sex and age of child


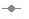
Grey estimate is additionally adjusted for maternal age, maternal educational level and maternal ethnicity.

**
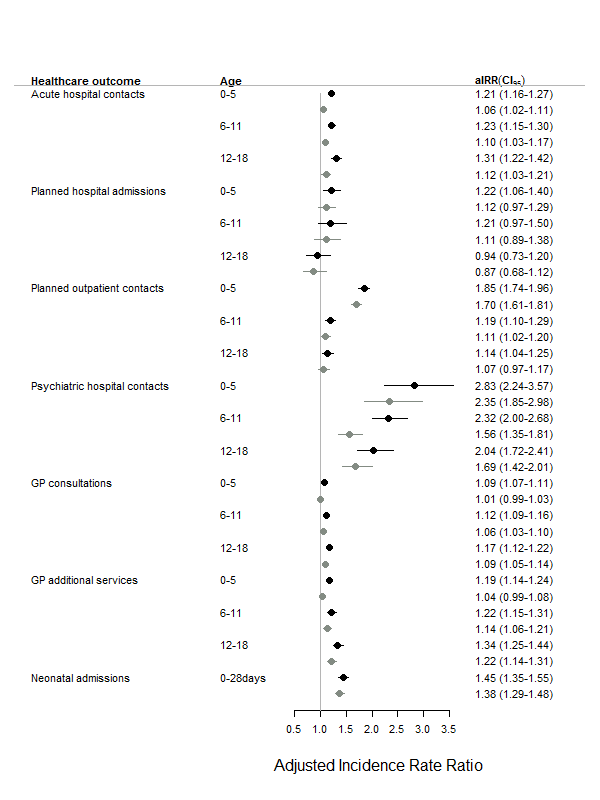
**

**Figure S7: Sensitivity analysis restricting the study population to only singleton first-born children: Incidence rate ratios for healthcare contacts among children with prenatal heavy alcohol exposure compared to reference children**

Study sample size: 3451 exposed, 603,341 reference children


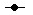
Black estimate is adjusted for year, sex and age of child


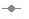
Grey estimate is additionally adjusted for maternal age, maternal educational level and maternal ethnicity.


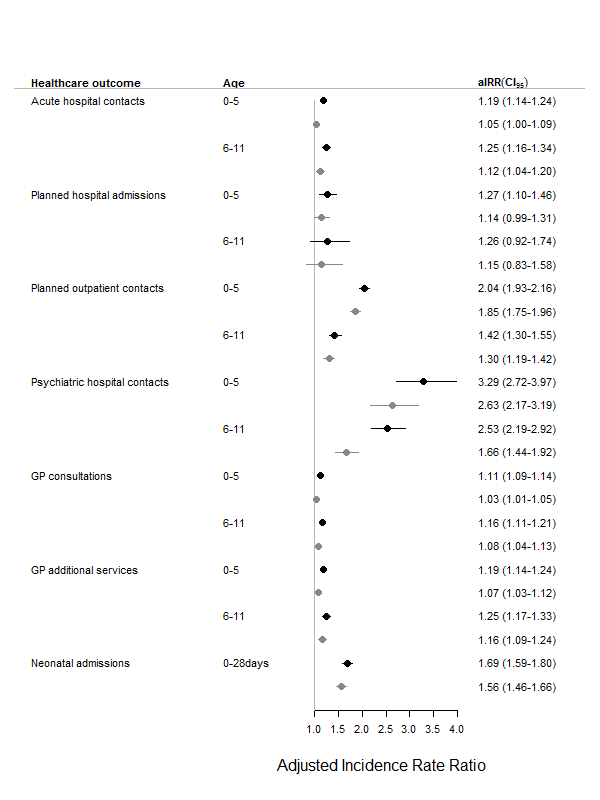


**Figure S8: Sensitivity analysis restricting the study period to 2008-2020: Incidence rate ratios for healthcare contacts among children with prenatal heavy alcohol exposure compared to reference children**

Study sample size: 3481 exposed, 755,974 reference children

Please note: Since the analysis is restricted to 2008-2020, the study population has a maximum age of 13 years for general practice outcomes and of 14 years for hospital outcomes, why estimates are only presented for ages 0-5 and 6-11.


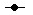
Black estimate is adjusted for year, sex and age of child


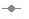
Grey estimate is additionally adjusted for maternal age, maternal educational level and maternal ethnicity.

**
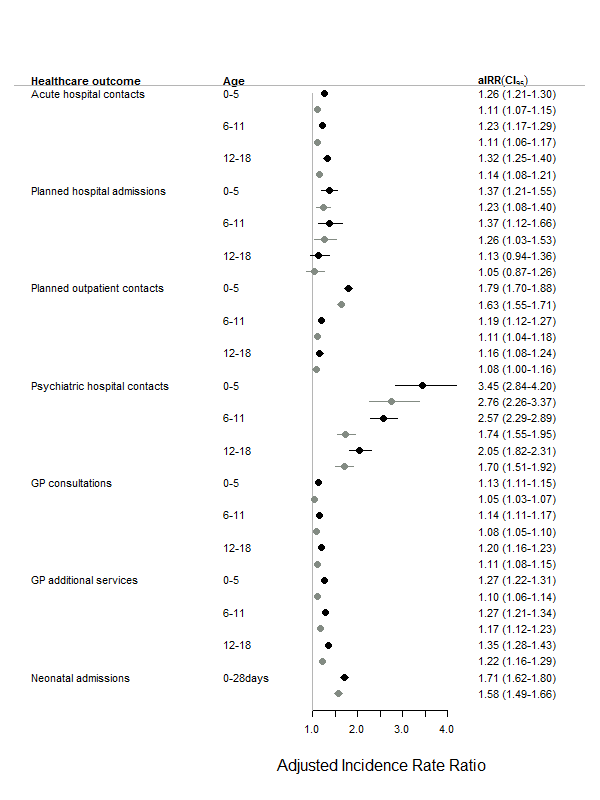
**

**Figure S9: Sensitivity analysis excluding exposure-defining diagnoses given to the child: Incidence rate ratios for healthcare contacts among children with prenatal heavy alcohol exposure compared to reference children**

Study sample size: 5271 exposed, 1,452,691 reference children. Note: This entails reclassifying 627 exposed children as reference children.


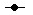
Black estimate is adjusted for year, sex and age of child


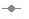
Grey estimate is additionally adjusted for maternal age, maternal educational level and maternal ethnicity.


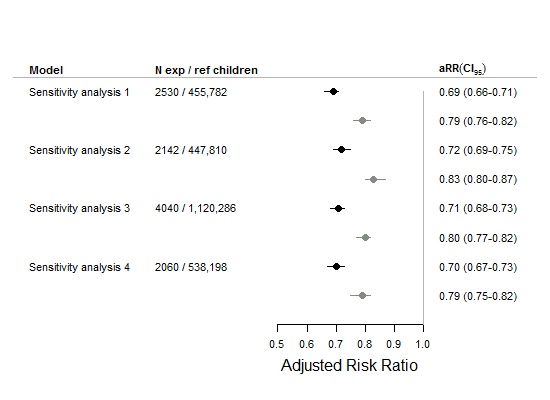


**Figure S10: Risk ratios from four sensitivity analyses for participation in above five preventive child health exams for children with heavy alcohol exposure compared to reference children, among children with full follow-up**

**Sensitivity analysis 1**: Restricting the study population to only singleton first-born children.

**Sensitivity analysis 2**: Restricting the study period to 2008 and onwards.

**Sensitivity analysis 3**: Excluding from the exposure definition diagnoses given to the child.

**Sensitivity analysis 4**: Restricting the study population to children born at full-term (gestational age 39+0-40+6)

**Abbreviations**: RR: Risk ratio, CI: Confidence interval


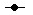
Black estimate is adjusted for year, sex and age of child


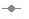
Grey estimate is additionally adjusted for maternal age, maternal educational level and maternal ethnicity.

**Supplementary material reference list**

[1] Centers for Disease Control and Prevention. Alcohol-related ICD codes [Internet]. Division of Population Health , National Center for Chronic Disease Prevention and Health Promotion , Centers for Disease Control and Prevention; 2020 July [cited 2023 Oct 05]. Available from: https://www.cdc.gov/alcohol/ardi/alcohol-related-icd-codes.html Date accessed: June 28, 2024.

[2] The Danish Health Data Authority. Status på alkoholområdet i sundhedssektoren: Registeranalyse af alkoholrelaterede hospitalskontakter, offentligt finansieret alkoholbehandling, dødsfald samt medicinsk alkoholbehandling blandt den danske befolkning for perioden 2014-2018 [Internet]. Copenhagen, Denmark: SDS; 2021 April. Available from: <https://sundhedsdatastyrelsen.dk/-/media/sds/filer/find-tal-og-analyser/sygdomme-og-behandlinger/alkoholstatistik/status_alkoholomraadet.pdf> Date accessed: June 28, 2024.

[3] Danish Pediatric Society. Fetal Alcohol Spectrum Disorders: FASD Retningslinje [Internet]. Denmark: DPS; 2021 Jan. Available from: <https://paediatri.dk/images/dokumenter/Retningslinjer_2021/DPS_FASD_Retningslinje_01.2021-komprimeret.pdf> Date accessed: June 28, 2024.

[4] Centers for Disease Control and Prevention. Indicator ICD-10-CM Codes: Drug use hospital data [Internet]. National Center for Health Statistics, Centers for Disease Control and Prevention; 2023 March. Available from: <https://www.cdc.gov/nchs/dhcs/drug-use/icd10-codes.htm> Date accessed: June 28, 2024.

[5] Burns L, Mattick RP, Cooke M. The use of record linkage to examine illicit drug use in pregnancy. Addict Abingdon Engl. June 2006;101(6):873–82.

[6] Toftdahl NG, Nordentoft M, Hjorthøj C. Prevalence of substance use disorders in psychiatric patients: a nationwide Danish population-based study. Soc Psychiatry Psychiatr Epidemiol. Jan 2016;51(1):129–40.

[7] United Nations Educational, Scientific and Cultural Organization (CA). ISCED: International Standard Classification of Education [Internet]. Available from: <https://uis.unesco.org/sites/default/files/documents/international-standard-classification-of-education-isced-2011-en.pdf> Date accessed: June 28, 2024.

[8] Højsgaard S, Halekoh U, Yan J, Ekstrøm CT. The R Package geepack, title: “Generalized Estimating Equation Package” [Internet]. Version 1.3.11, June 2024. Available from: <https://cran.r-project.org/web/packages/geepack/geepack.pdf> Date accessed: June 28, 2024.

[9] Halekoh, U, Højsgaard S, Yan J. The R Package geepack for Generalized Estimating Equations. Journal of Statistical Software. Jan 2006:Vol. 15(2):1–11.
